
JOURNAL INFORMATION
==============================
NLM Title Abbreviation: Wirtschaftsdienst
Journal ID: Wirtschaftsdienst
NLM Journal ID: 101086612

Wirtschaftsdienst (Hamburg, Germany : 1949)

ISSN: 0043-6275

ARTICLE INFORMATION
==============================
PMCID: PMC8536894
PMID: 34720231
DOI: 10.1007/s10273-021-3014-2
Article ID: 3014
Article version: 1
Subjects: Kurz Kommentiert

Ausbildungsmarkt: Risiko für die Fachkräftesicherung

Training market: Risk for securing skilled labour

Leber Ute Ute.Leber@iab.de

grid.425330.3 0000 0001 1931 2061 Institut für Arbeitsmarkt- und Berufsforschung (IAB), Regensburger Straße 104, 90478 Nürnberg, Deutschland
Electronic publication date: 2021 Oct 23
Print publication date: 2021
Volume: 101
Issue: 10
First page: 750
Last page: 750
Copyright: © Der/die Autor:in 2021
License: Open Access: Dieser Artikel wird unter der Creative Commons Namensnennung 4.0 International Lizenz veröffentlicht (https://creativecommons.org/licenses/by/4.0/deed.de).
Open Access wird durch die ZBW — Leibniz-Informationszentrum Wirtschaft gefördert.
License URL: https://creativecommons.org/licenses/by/4.0/

issue-copyright-statement: © The Author(s) 2021

==============================

DOI: 10.1007/s10273-021-3014-2

Ausbildungsmarkt

### Risiko für die Fachkräftesicherung

Die Berufsausbildung spielt nicht nur für junge Erwachsene eine wichtige Rolle beim Übergang in das Erwerbsleben, sie ist auch für die Betriebe zentral, um ihren Personalbedarf zu decken. Umso problematischer ist es, dass in den vergangenen Jahren immer mehr Ausbildungsplätze nicht besetzt werden konnten. Blieb bis zur Mitte der 2000er Jahre noch knapp jede zehnte Lehrstelle vakant, war zuletzt mehr als ein Viertel hiervon betroffen. Um Fachkräfteengpässe in der Zukunft zu vermeiden, gilt es, dem jetzt entgegenzusteuern. Verantwortlich für den Anstieg ist zunächst die demografische Entwicklung: Die Zahl der Schulabgänger:innen ist in den vergangenen Jahren deutlich gesunken, sodass weniger potenzielle Bewerber:innen auf dem Ausbildungsmarkt zur Verfügung stehen. Hinzu kommt, dass sich immer mehr junge Menschen für ein Studium an Stelle einer Lehre entscheiden. Zwar ist die Zahl derer, die eine Ausbildung beginnen, nach wie vor höher als die der Studienanfänger:innen, doch haben sich die Relationen zugunsten des Studiums verschoben. Neben dem quantitativen Rückgang spielt auch die Qualität der Schulabgänger:innen eine Rolle. So klagen Betriebe vielfach, dass Bewerber:innen nicht über ausreichende fachliche oder soziale Kompetenzen verfügen, die Voraussetzung für die Aufnahme und den erfolgreichen Abschluss einer Berufsausbildung sind.

Unter dem Eindruck der COVID-19-Pandemie ist aktuell ein weiterer Rückgang der Bewerber:innen festzustellen. Dies hat mit dem Wegfall von vielen Angeboten der Berufsorientierung und von Praktika zu tun, die eine wichtige Rolle bei der Entscheidung für eine Ausbildung spielen. Zu beobachten ist aber auch, dass sich ein Teil der Jugendlichen in wirtschaftlichen Krisenzeiten angesichts der Unsicherheit über die zukünftige Entwicklung für alternative Bildungswege entscheidet und beispielsweise ein Studium an Stelle einer Ausbildung aufnimmt.

Auffällig ist, dass sich das Phänomen unbesetzter Ausbildungsplätze stark zwischen einzelnen Segmenten der Wirtschaft unterscheidet. Zunächst haben vor allem Kleinstbetriebe mit Besetzungsproblemen zu kämpfen. Eine Ausbildung in einem kleinen Betrieb erscheint vielen Jugendlichen wenig attraktiv, weil z. B. die Aufstiegs- oder Verdienstmöglichkeiten geringer als in einem Großunternehmen sind. Zudem suchen viele kleine Firmen ihr Personal auf anderem Wege als größere Betriebe – mit der Folge, dass vorhandene Ausbildungsplatzangebote potenziellen Bewerber:innen oftmals gar nicht bekannt sind.

Weit überdurchschnittliche Besetzungsschwierigkeiten haben Betriebe aus dem Baugewerbe, dem Gaststätten- und Hotelgewerbe sowie dem Lebensmittelhandwerk. Auch hier handelt es sich vielfach um Kleinbetriebe, die im Wettbewerb um Lehrlinge Nachteile gegenüber Großbetrieben haben. Hinzu kommen in den genannten Branchen aber auch weitere Faktoren wie ungünstige Arbeitszeiten, welche die Besetzung von Lehrstellen zusätzlich erschweren. Schließlich gibt es nennenswerte regionale Unterschiede. Hohe Anteile an unbesetzten Ausbildungsplätzen finden sich in Ostdeutschland, aber auch in Teilen Bayerns und Baden-Württembergs. Vor allem Betriebe in ländlichen Räumen tun sich bei der Suche nach Lehrlingen schwer. Besonders problematisch wird es, wenn mehrere der genannten Risikofaktoren der Nichtbesetzung von Ausbildungsplätzen aufeinandertreffen. Deutlich wird dies beispielsweise im kleinstbetrieblichen Segment in Ostdeutschland, wo den Ergebnissen des IAB-Betriebspanels zufolge zuletzt rund die Hälfte der angebotenen Lehrstellen unbesetzt blieb.

Viele Betriebe setzen auf das Angebot an Zusatzleistungen, um die Attraktivität ihrer Ausbildung zu erhöhen. Dabei handelt es sich etwa um Beihilfen zur Mobilität, die gerade in wenig bevölkerten Gebieten bedeutsam sein können. In manchen Fällen führen anhaltende Schwierigkeiten bei der Besetzung von Lehrstellen aber auch dazu, dass sich Betriebe aus der Ausbildung zurückziehen. So ist im kleinstbetrieblichen Segment ein Rückgang der Ausbildungsbeteiligung auszumachen, der mit den Rekrutierungsschwierigkeiten in diesem Bereich korrespondiert.

Um dem Problem unbesetzter Lehrstellen zu begegnen, ist es wichtig, Angebot und Nachfrage auf dem Ausbildungsmarkt besser als bislang zusammenzuführen. Trotz der gestiegenen Zahl an nichtbesetzten Ausbildungsplätzen gibt es nach wie vor viele junge Menschen, die bei der Suche nach einer Lehrstelle unversorgt bleiben. Erfolgversprechend erscheinen hier Ansätze, die ein beiderseitiges Kennenlernen ermöglichen und Jugendliche nicht nur an die Ausbildungsinhalte im Betrieb heranführen, sondern auch den Arbeitgebenden Einblicke in die Stärken und Schwächen der jungen Menschen gewähren. Zugleich müssen aber auch verstärkt Maßnahmen getroffen werden, um das Interesse der Jugendlichen für eine betriebliche Ausbildung zu fördern. Entsprechende Initiativen von Betrieben, Arbeitsagenturen und Kammern sind hier ein wichtiger Schritt.

Ute Leber
Institut für Arbeitsmarkt- und Berufsforschung (IAB)
Ute.Leber@iab.de

© Der/die Autor:in 2021. Open Access: Dieser Artikel wird unter der Creative Commons Namensnennung 4.0 International Lizenz veröffentlicht (creativecommons.org/licenses/by/4.0/deed.de).

Open Access wird durch die ZBW – Leibniz-Informationszentrum Wirtschaft gefördert.
